# Safety and efficacy of the swift microwave device in patients with mild‐to‐moderate onychomycosis: Protocol of an open‐label, randomized, dose‐finding pilot study

**DOI:** 10.1002/ski2.455

**Published:** 2024-10-10

**Authors:** Aditya K. Gupta, Elizabeth A. Cooper, Tong Wang

**Affiliations:** ^1^ Division of Dermatology Department of Medicine, Temerty Faculty of Medicine University of Toronto Toronto Ontario Canada; ^2^ Mediprobe Research Inc. London Ontario Canada

## Abstract

**Background:**

Onychomycosis is a common fungal nail disease with a prevalence rate up to 14% in North America and 24% in Europe. The current treatment paradigm is limited by a high risk of disease recurrence, safety concerns for oral agents, and a low likelihood of patients achieving both clinical improvement and mycological cure. Recent advances in device‐based treatments have allowed for the direct targeting of the infection site that bypasses drug resistance mechanisms while minimizing systemic side‐effects. The Swift^®^ System is a microwave device that has demonstrated therapeutic potential in treating skin (e.g. verrucae vulgaris, actinic keratosis) and nail infections.

**Methods:**

We report the protocol of an open‐label, randomized, pilot study that will be conducted at a single Canadian center. Our primary objective is to evaluate the safety and efficacy of microwave treatment (Swift^®^ System, Emblation Ltd, Scotland, U.K.), administered at three different dosing regimens, in 45 patients with mild‐to‐moderate distal subungual onychomycosis. Our secondary objective is to identify an optimal dosing regimen, if any, to better inform the conduct of a future pivotal trial. Patients will be randomized (1:1:1) to undergo either 9 treatment sessions (5 weekly sessions plus 4 monthly sessions), 7 treatment sessions (3 sessions every 2 weeks plus 4 monthly sessions), or 12 treatment sessions every 2 weeks. At each session microwave energy will be applied in 3‐s intervals at 7–9 Watts, repeated up to 5 times at each treatment position on the nail. Overlapping treatment positions are used to ensure sufficient coverage of the infected area. Patients will be enrolled in the trial over a 12‐month period. Efficacy will be evaluated based on visual improvement and mycology testing results. Adverse events will be recorded throughout the entire study period.

**Discussion:**

This study will be the first to report on the safety and efficacy of microwave treatment in onychomycosis patients in a trial setting; recruitment is ongoing.

**Trial registration:**

ClinicalTrials.gov, NCT05674747.

## 1 BACKGROUND

Onychomycosis, or tinea unguium, is the most common cause of nail disorders worldwide accounting for at least half of nail dystrophies seen in the clinic.^1^ This chronic, slow‐progressive, fungal nail disease is more prevalent among the elderly and in individuals living with diabetes and peripheral vascular disease.^1^, ^2^ Clinical manifestations include nail thickening, brittleness, discolouration and nail plate separation (onycholysis), which may be accompanied by localised pain and numbness impairing ambulation.^1^

The most common presentation of onychomycosis is infection that originates at the hyponychium or the lateral nail fold which then progresses proximally invading the stratum corneum of the nail bed,^3^ known as distal subungual onychomycosis (DSO). Dermatophytes, a group of filamentous fungi with the ability to degrade keratin, are frequently identified as the causative organisms in onychomycosis patients; in particular, *Trichophyton* (i.e., *T*. *rubrum* and *T*. *mentagrophytes*) is the predominant dermatophyte found in North America.^4^

Although several topical and oral treatment options exist,^5^ there remains an unmet need for treatment that achieves both clinical and mycological cure (i.e., normal‐appearing nail with the complete eradication of the underlying fungal infection) and treatment that reduces the risk of disease recurrence. Device‐based treatments, such as lasers, photodynamic therapies and microwaves, offer a promising potential in directly targeting the site of infection with minimal systemic side‐effects risk.^6^ Furthermore, device‐based modes of treatment can bypass dermatophyte antifungal resistance mechanisms towards oral and topical agents (e.g., terbinafine) as recently reported.^7^, ^8^ Despite these advantages, clinical reports available for devices remain limited other than lasers where high‐quality data and results are still inconsistent. The absence of conclusive data for any single treatment to date highlights the need for broadening the medical armamentarium to address all the challenges posed by onychomycosis.

Microwaves as a treatment modality for fungal infections offer potential as a unique approach as they use water within the tissue to generate heat. Microwaves do not generate heat the way lasers do—by direct absorption of photons. Instead, microwaves create rapidly oscillating electric fields that cause water, ions and other small polar molecules to rotate or move, which in turn generates heat. This means that microwaves can have good penetration, are non‐ionizing (i.e., do not damage proteins and DNA directly), and still can rapidly heat tissues. This permits rapid heating of infected nail plates, even if dystrophic nails are thick and opaque. Lasers, in contrast, rely on thermal diffusion of heat generated at the nail surface to reach dermatophytes in and below the nail plate.

Microwave treatment, through its multimodal mechanisms of action, has previously demonstrated antifungal effects in vitro.^6^ In addition to locally induced hyperthermia leading to protein denaturation and fungal cell death, microwave energy may disrupt the ability of dermatophytes to evade natural defence mechanisms and immune responses of the nail.^6^, ^9^ Previous studies have demonstrated favourable efficacy of the Swift^®^ microwave device in patients with plantar warts, including in patients with multiple prior failed treatments and warts lasting for ≥2 years^10^, ^11^ In a case report, a patient presenting with onychomycosis complicated by dermatophytoma (i.e., compacted fungal elements recalcitrant to treatment) demonstrated significant clinical improvement after undergoing six treatment sessions.^12^

The Swift^®^ System is a microwave device that has been cleared by the US FDA (510(k) number: K181941), Health Canada for general dermatology and podiatry use (licence number: 98352) and is CE (Conformité Européenne) marked, allowing it to be sold within the European Union in compliance with European medical device regulations. In this trial, we sought to evaluate the safety and efficacy of the Swift^®^ microwave treatment, administered at three different dosing regimens, in Canadian patients with mild‐to‐moderate onychomycosis. To our knowledge, the present trial is the first to evaluate the safety and efficacy of microwave treatment in onychomycosis patients, utilising both the US FDA recommended endpoint and mycological testing.

## 2 METHODS/DESIGN

In this open‐label, randomized, pilot study, we aim to evaluate the safety and efficacy of microwave treatment (Swift^®^ System, Emblation Ltd.) for mild‐to‐moderate onychomycosis at a single Canadian dermatology clinic (Mediprobe Research Inc.). A total of 45 subjects will be enroled into the study and efficacy will be evaluated primarily based on the ‘temporary increase of clear nail’ in accordance with the US FDA recommendation.^13^ Our secondary objective is to identify an optimal dosing frequency, if any, to better inform the conduct and design of a pivotal trial.

This pilot trial has been prospectively registered with ClinicalTrials.gov in January, 2023 (NCT05674747). The trial protocol, recruitment material and consent form has received Institutional Review Board (IRB) approval (Advarra, Inc., protocol number: FN1p, version: amendment 1, date: 19 May 2023). The trial will be conducted in adherence to the International Conference on Harmonisation Good Clinical Practice (ICH GCP) and the International Organisation for Standardisation (ISO) Clinical Investigation of Medical Devices on Human Subjects‐Good Clinical Practice; local and national regulations including the Health Canada Medical Device Regulations will be followed. Any changes in study design will require formal protocol amendments approved by Advarra IRB. The principal investigator is primarily responsible for the design and conduct of the trial, preparations of the study protocols including amendments and managing the trial site. The support service personnel will prepare case report forms and conduct imaging analyses and statistical analyses. Reporting of the trial protocol herein follows the recommendation by Thabane and Lancaster^14^ and is in accordance with SPIRIT (Standard Protocol Items: Recommendations for Interventional Trials) guideline.^15^, ^16^

### 2.1 Study participants

Adult patients presenting with DSO of one hallux, with 20%–75% involvements of the nail area, nail thickness of ≤3 mm and no area of infection <3 mm from the proximal nail fold, will be enroled and allocated to treatment. Inclusion and exclusion criteria are summarised in Table 1. No eligibility waivers will be permitted.

**TABLE 1:** Patient eligibility criteria.

Inclusion criteria
Provide written informed consent Aged ≥18 years Not pregnant or breastfeeding Subject presenting with distal subungual onychomycosis of one hallux Mycology‐positive diagnosis confirmed by the detection of a dermatophyte One hallux as a treatment target with 20%–75% nail area affected One hallux as a treatment target with a thickness of ≤3 mm No area of infection <3 mm from the proximal nail fold No more than 4 toenails showing visual signs of onychomycosis including the treatment target Subject agrees not to take any oral or topical antifungal treatments for the duration of the study Subject able to undergo study assessments

Exclusion criteria
Subject presenting with proximal subungual onychomycosis or superficial white onychomycosis Hallux designed as the treatment target shows disease involvement of the lunula Non‐onychomycotic dystrophy presenting as parallel lines, small pinpoint depressions, brown spots, black or brown linear streaks, complete yellowing of all nails without textual changes, green debris below the nail or notches in the nail margin Causative fungal agent belonging to rare species or non‐fungal organisms such as non‐dermatophyte mould, *Candida*, or bacteria ‘Spike’ of onychomycosis extending to <3 mm away from the eponychium Presence of dermatophytoma Type I or II diabetes mellitus Peripheral vascular disease Recurrent cellulitis Lymphatic insufficiency Immunocompromised Severe moccasin tinea pedis Any uncontrolled condition which, in the opinion of the investigator, may put the subjects at undue risk of adverse effects during the study Exhibit an increased risk of bacterial infections due to skin breakdown caused by a fungus Co‐morbidities known to induce nail changes including psoriasis and lichen planus Trauma due to ill‐fitting shoes, running or overly‐aggressive nail care Regularly scheduled podiatric/nursing nail care History of recent toenail surgery with residual disfigurement Recent topical onychomycosis treatments ≥3 months prior to study treatment or oral onychomycosis treatments ≥6 months prior to study treatment Implantable cardioverter defibrillator, pacemaker or other implantable devices Metal implants at or near the treatment site History of intolerance or allergy to microwave therapy History of any topical metallic or ionic treatment within the last 6 months Current participation of another non‐observational trial or within the last 30 days

**FIGURE 1:**
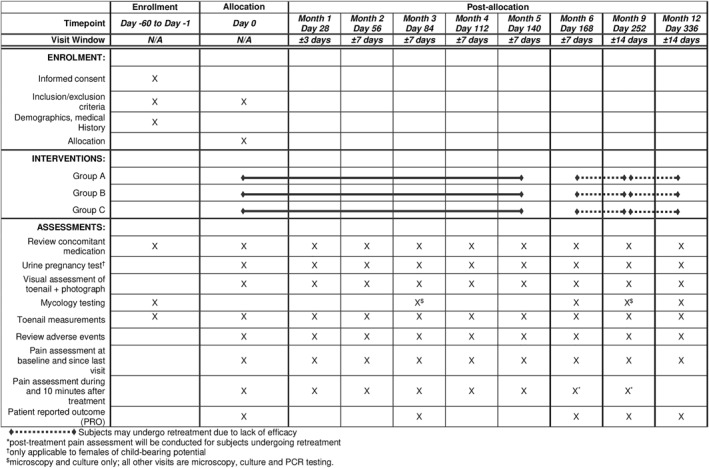
Summary of study schedule (SPIRIT 2013 figure).^15^, ^16^

Patients will be evaluated at enrolment, which includes medical history, concomitant medications (prescriptions, over‐the‐counter medications, supplements), onychomycosis disease duration and treatment history, and toenail measurements (i.e. nail thickness, total affected nail surface area and lowest distance between the affected nail area to the proximal nail fold) (Figure 1); Flyers/posters, online and radio advertisements will be used to increase enrolment. Once a patient provides written informed consent and meets the eligibility criteria, one affected hallux will be sampled for mycology testing (i.e., fungal culture, polymerase chain reaction [PCR] and direct potassium hydroxide [KOH] microscopy). Patients testing PCR and/or fungal culture positive for dermatophytes will be eligible to join the study.

In the case that a subject presents with onychomycosis affecting both halluces, then both halluces will be measured and sampled for mycology testing as described above. If both halluces test positive for dermatophytes, then the hallux with the larger affected nail surface area within the inclusion limit will be designated as the treatment target. If a dermatophyte is not detected at screening, then a repeat sample may be taken during a subsequent visit; in the case that a repeat sample tests positive for dermatophyte, then the subject will qualify for the study. Additionally, up to three other toenails may be designated as ‘non‐target’ toenails at the investigator's discretion and will receive the same treatment.

### 2.2 Study intervention

On day 0, patients will be randomized (1:1:1) to one of three treatment groups (Groups A–C) based on a computer‐generated randomisation schedule generated prior to the start of the study (Figure 1). Group A will undergo nine treatment sessions, which includes the first session at day zero followed by four weekly sessions then four monthly sessions. Group B will undergo 7 treatment sessions, which includes the first session at day 0 followed by sessions every 2 weeks for 2 sessions then 4 monthly sessions. Group C will undergo 12 treatment sessions, which includes the first session at day 0 followed by sessions every 2 weeks.

Non‐responders at the end of the month 6 visit (day 168) or month 9 visit (day 252) as judged by the US FDA recommended endpoint (i.e., “temporary increase of clear nail”) will be given the option to undergo retreatment once per month for 3 months up to month 9 or month 12, respectively (Figure 1).^13^ Subjects who do not wish to undergo retreatment will be asked to return for month 9 and month 12 visits.

Tinea pedis is a common co‐infection seen in onychomycosis patients^17^; in this study, patients presenting with concomitant tinea pedis, at the investigator's discretion, may be provided with topical antifungal agents for treating affected skin areas. Toenails will be strictly avoided. No local anaesthetic, petroleum jelly nor metallic‐based topical treatments (e.g., aluminium chloride, silver nitrate) are to be used in the toenail areas during this study. Concomitant use of oral antifungal agents is not permitted.

### 2.3 Treatment procedures

At enrolment, one affected hallux will be designated as the treatment target; up to 3 additional ‘non‐target’ toenails may also receive treatment. Prior to treatment, all visual and photographic assessments will be performed, in addition to sampling for mycology testing, as per the study schedule (Figure 1).

During each treatment session, toenails will be prepared by the clinician. Nails will be soaked in warm water for a minimum of 5 min to aid hydration and microwave energy absorption thereby generating heat in the nail plate and nail bed; then they will be cleaned with an antiseptic solution and allowed to dry. Microwave energy will be applied locally at a pre‐specified dose of 7–9 W for up to 3 s (i.e., one “burst”); treatment may be initiated at 9 W for up to 3 s with further adjustments of power and time settings depending on patient tolerability. Up to 5 additional bursts, spaced approximately 5‐s apart and subject to patient tolerability, may be administered in the same location and then repeated at a new location to ensure complete coverage of the nail surface. The affected nail area for treatment is chosen by the clinician administering the microwave energy and is concentrated on the proximal margin of discolouration and then distally as needed. The dose, duration and number of repetitions from each session will be recorded.

Investigators will follow the operation manual of the microwave device (Swift^®^ System, Emblation Ltd.). A new disposable tip will be fitted to the applicator probe used during each treatment session of up to 15 min from the first delivery of microwave energy. Ambient operating temperature should be between 10 and 30°C.

### 2.4 Study assessments

All enroled subjects will be assessed until month 12 for the evaluation of efficacy and safety endpoints, including interim treatment visits (Figure 1). Clinical response will be evaluated based on in‐person assessments and photographic images of the treated hallux, both in terms of the affected nail area and nail length. Mycology testing will be performed at baseline, day 84 (month 3), day 168 (month 6), day 252 (month 9) and day 336 (month 12).

Incidence of adverse events (AEs) will be recorded throughout the entire study period; each AE will be graded based on severity (mild, moderate, severe) and relatedness to the study intervention. The investigator will deem an AE as ‘expected’ if it's consistent with previous reports in the literature,^10^, ^11^, ^18^ which includes local application site reactions such as pain, bruising, heat sensitivity and tingling. Burns, blisters, or nail disfigurement will be considered as ‘unexpected’ AEs. Any new clinically relevant findings deemed by the investigator will be reported as an AE. Patients will be asked to report their pain level—categorised as none, mild, moderate or severe—before treatment, during treatment, and 10‐min post‐treatment. Additionally, patient‐reported outcomes (PROs) will be recorded at pre‐specified timepoints of which patients will be asked to rate (a) how their daily activities are affected, (b) how their lifestyle activities (e.g. sports, hobbies) are affected and (c) how their mood is affected.

Subjects who wish to discontinue from treatment will be asked to complete all remaining study assessments as planned. The principal investigator may withdraw a subject from the study if there is significant protocol non‐compliance, AEs or other medical conditions such that the continued study participation would not be in the interest of the subject, or subject meeting the exclusion criteria during the study period. If a subject fails to return for 2 scheduled visits, and is unable to be contacted (through multiple telephone calls and letters), the site staff will make a reasonable effort to ascertain reason(s).

### 2.5 Primary endpoints

The co‐primary endpoints include (a) an acceptable safety and tolerability profile at the end of month 6 (day 168 ± 7 days) defined by treatment adherence with minimal mild‐to‐moderate treatment‐related AEs (expressed as frequencies per category), which resolve before further treatments with no sequelae, and (b) proportion of subjects achieving the following endpoints at month 3 (day 84 ± 7 days) and month 6 (day 168 ± 7 days): month 3—US FDA definition of ‘temporary increase in clear nail’ with evidence of nail improvements defined as an increase in clear nail measured in distance from the proximal nail fold or an increase of the clear nail area; month 6—achieving either (1) ≥ 6 mm increase in clear nail measured in distance from the proximal nail fold, (2) an additional 60 mm^2^ of the treated nail area achieving clearance with evidence of outward growth or (3) complete clearance if less than 6 mm of the distal nail was involved at baseline.

### 2.6 Secondary endpoints

Secondary safety endpoints include (a) the maintenance of an acceptable safety and tolerability profile beyond the end of month 6 (day 168 ± 7 days) for patients undergoing retreatment defined by treatment adherence with minimal mild‐to‐moderate treatment‐related AEs (expressed as frequencies per category), which resolve before further treatments with no sequelae, (b) frequency of ‘expected’ AEs, (c) incidence of AEs that differentiate ‘target’ and ‘non‐target’ toenails, (d) comparison of pain levels during and after treatment and (e) comparison of pain levels as a function of the number of treatments and treatment frequency.

Secondary efficacy endpoints include (a) proportion of subjects achieving at least one of the two criteria—a ≥3 mm increase in clear nail measured in distance from the proximal nail fold or a ≥30% increase in the total nail area achieving clearance with evidence of outward growth —at the end of month 6 (day 168 ± 7 days), (b) proportion of subjects achieving improved PROs, (c–e) proportion of subjects achieving mycological cure (negative mycology testing results) achieved at the end of, (c) month 6 (day 168 ± 7 days), (d) month 9 (day 252 ± 14 days) and (e) month 12 (day 336 ± 14 days) and (f,g) proportion of subjects achieving effective cure (≤10% of the total nail area affected with mycological cure) achieved at the end of (f) month 6 (day 168 ± 7 days), (g) month 9 (day 252 ± 14 days) and (h) month 12 (day 336 ± 14 days).

### 2.7 Data management

Data collection is the responsibility of the clinical trial staff under the supervision of the principal investigator. The principal investigator is responsible for ensuring the accuracy, completeness, legibility and timeliness of the data reported. Case report forms in a paper format will be used for this study. Forms will be reviewed against source documentations. Any changes or corrections will be tracked on paper. The investigator will permit inspections of the trial site and documentation by clinical research personnel, as well as external auditors or representatives from regulatory authorities.

Subject privacy and confidentiality will be strictly held in trust by the principal investigator and trial personnel. This includes the study protocol, documentation, data and all other information generated. A unique number will be assigned to each subject at the start of the trial and this, with the subject's initials, may be used to identify the subject. The investigator will keep a list of identification codes or initials used for each subject along with the unique number assigned. IRB representatives, or other regulatory bodies may inspect all documents and records required to be maintained by the investigator.

### 2.8 Data analysis

As a pilot study, sample size calculations were not performed; a sample size of 15 patients per treatment group was selected to provide sufficient preliminary data on treatment safety, tolerability and efficacy in onychomycosis patients.

The per‐protocol and intention‐to‐treat populations will be included for safety and efficacy analyses. Descriptive statistics will be conducted for subject demographic and baseline parameters including sex, Fitzpatrick skin type (I‐VI) and fungal species. All AEs will be presented by description, category, frequency, severity and causality; differences in incidence between Groups A and C will be tabulated. Statistical analyses will be performed if there are sufficient data points available.

Statistical analyses for the difference in proportion of subjects achieving a pre‐specified efficacy endpoint between treatment groups will be conducted using the Chi‐square test; *p* < 0.05 will be considered statistically significant. Nail measurements will be converted to the mean increase in clear nail, both in terms of clear nail length from the proximal nail fold and clear nail surface area. The mean increase in clear nail, mean increase in clear nail as a function of the number of treatments and energy dose administered, mean increase in clear nail as a function of the number of visits attended and mean pain scores at treatment visits will be analysed using ANOVA or the Kruskal–Wallis test depending on data distribution; *p* < 0.05 will be considered statistically significant. Additional correction measures will be performed at the statistician's discretion.

## 3 FUTURE DIRECTIONS

In this pilot study, our efficacy endpoint for microwave treatments was designed primarily based on the US FDA recommendation of ‘temporary increase in clear nail in patients with onychomycosis’ (i.e., a cosmetic endpoint).^13^ As an extension, mycology testing will be conducted to indicate the eradication of the underlying fungal infection (i.e., mycological cure) and matched to patients achieving concurrent improvements in clear nail (i.e., effective cure). To improve the comparability of our visual assessment results, the Onychomycosis Severity Index (OSI), a standardized metric system proposed by Carney et al.,^19^ should be considered for future trials. The OSI incorporates three visual parameters: (a) percent of nail area involvement, which translates to a score of 1–5 (1: <10%, 2: 11%–25%, 3: 26%–50%, 4: 41%–75%, 5: >75%), (b) proximity of the affected area to the nail matrix whereby the nail plate is divided transversely into 5 sections, a score (1–5 [distal‐proximal]) is assigned based on the location of the most proximal edge of the affected nail area, and (c) presence of dermatophytoma and/or subungual hyperkeratosis ≥2 mm that adds an additional 10 points.^20^ The final OSI score is calculated by multiplying parameter (a) and (b), with or without the additional 10 points as per parameter (c), for a maximum of 35 points (0: clinically normal‐appearing nail, <5: mild onychomycosis, 6–15: moderate onychomycosis, >15: severe onychomycosis).^20^ Psychological burden impairing the quality of life is an important burden experienced by patients with onychomycosis^21^; a standardized questionnaire (e.g., OnyCOE‐t^TM^, NailQoL) should also be considered for future trials. The OnyCOE‐t^TM^ questionnaire proposed by Potter et al. contains 33 items divided into six domains: toenail symptom, appearance problems, physical activity problems, overall problems, stigma and treatment satisfaction.^22^ The average score from each domain is calculated and transformed into a scale of 0–100 (no impairment to severe impairment).

## 4 TRIAL STATUS

Initial IRB approval of the study protocol was obtained in December 2022 and enrolment began in March 2023. Study inclusion criteria was expanded from ‘20%–50% nail area affected’, and ‘nail thickness ≤1.5 mm’ to ‘20%–75% nail area affected’ and ‘nail thickness ≤3.0 mm’, which were covered in the first protocol amendment to increase the recruitment rate. Mycology testing at enrolment was revised to include PCR testing allowing for a positive dermatophyte detection by PCR and/or fungal culture. The revised protocol received IRB approval in June 2023. Enrolment began in March 2023 and is complete as of May 2024. A total of 39 patients were enroled due to difficulty in confirming a positive dermatophyte infection. Study visits will continue through 2024, with interim data available in December 2024 and anticipated trial completion by May 2025.

## CONFLICT OF INTEREST STATEMENT

Elizabeth A. Cooper and Tong Wang declare that they have no competing interests that are relevant to the content of this article. Aditya K. Gupta is the principal investigator and provides consultancy for Emblation Limited.

## AUTHOR CONTRIBUTIONS

**Aditya K. Gupta**: Conceptualisation (lead); methodology (equal); project administration (lead); resources (lead); supervision (lead); writing—review and editing (equal). **Elizabeth A. Cooper**: Methodology (equal); writing—review and editing (equal). **Tong Wang**: Visualisation (lead); writing—original draft (lead).

## ETHICS STATEMENT

The study protocol and consent form have been approved by the Institutional Review Board at Advarra (Advarra Inc., Aurora, ON, Canada).

## PATIENT CONSENT

All patients will provide written informed consent prior to participating in this study.

## Data Availability

Research data are not found.
